# Autophagy Plays a Critical Role in ChLym-1-Induced Cytotoxicity of Non-Hodgkin’s Lymphoma Cells

**DOI:** 10.1371/journal.pone.0072478

**Published:** 2013-08-28

**Authors:** Jiajun Fan, Xian Zeng, Yubin Li, Shaofei Wang, Ziyu Wang, Yun Sun, Hongjian Gao, Guoping Zhang, Meiqing Feng, Dianwen Ju

**Affiliations:** 1 Department of Biosynthesis, School of Pharmacy, Fudan University, Shanghai, China; 2 Key Laboratory for Microbiological Engineering of Agricultural Environment, Ministry of Agriculture, College of Life Science, Nanjing Agricultural University, Nanjing, Jiangsu, China; 3 School of Medicine, Fudan University, Shanghai, China; 4 Institute of Biomedical Science, Fudan University, Shanghai, China; University of Barcelona, Spain

## Abstract

Autophagy is a critical mechanism in both cancer therapy resistance and tumor suppression. Monoclonal antibodies have been documented to kill tumor cells via apoptosis, antibody-dependent cellular cytotoxicity (ADCC) and complement-dependent cytotoxicity (CDC). In this study, we report for the first time that chLym-1, a chimeric anti-human HLA-DR monoclonal antibody, induces autophagy in Raji Non-Hodgkin’s Lymphoma (NHL) cells. Interestingly, inhibition of autophagy by pharmacological inhibitors (3-methyladenine and NH_4_Cl) or genetic approaches (siRNA targeting Atg5) suppresses chLym-1-induced growth inhibition, apoptosis, ADCC and CDC in Raji cells, while induction of autophagy could accelerate cytotoxic effects of chLym-1 on Raji cells. Furthermore, chLym-1-induced autophagy can mediate apoptosis through Caspase 9 activation, demonstrating the tumor-suppressing role of autophagy in antilymphoma effects of chLym-1. Moreover, chLym-1 can activate several upstream signaling pathways of autophagy including Akt/mTOR and extracellular signal-regulated kinase 1/2 (Erk1/2). These results elucidate the critical role of autophagy in cytotoxicity of chLym-1 antibody and suggest a potential therapeutic strategy of NHL therapy by monoclonal antibody chLym-1 in combination with autophagy inducer.

## Introduction

Lymphoma is one of the most common tumors in the world, causing almost 20 thousand deaths every year. Monoclonal antibodies have been reported to be an effective choice in lymphoma therapy in both animal models and clinical practice [1]. ChLym-1, a chimeric anti-HLA-DR monoclonal antibody in phase II clinical trials, shows more potent antilymphoma effects than Rituximab (anti-CD20 monoclonal antibody) in human NHL [2]–[4]. Previous study demonstrated that antilymphoma antibodies Rituximab and chLym-1 could cause cytotoxicity of NHL cells via apoptosis, antibody-dependent cellular cytotoxicity (ADCC) and complement-dependent cytotoxicity (CDC); however, the exact mechanisms involved in their tumor-killing effects still remain unclear [5].

Autophagy is a basic phenomenon in eukaryotes and a key ingredient in cell microenvironment maintenance [6]. It is induced when cells are lack of nutrients, deprived of growth factors and hypoxia [7]. Recent research reveals that autophagy can be induced by anti-tumor therapy and is significantly associated with therapy-induced cell death, acting as a “double-edged sword in tumor therapy [8], [9]. On one hand, inhibition of autophagy enhances the efficacy of drugs like 5-FU, Cetuximab, and Trastuzumab, indicating the cell protective role of autophagy in tumor therapy [10]–[12]. On the other hand, as to some other drugs like As_2_O_3_, autophagy can induce apoptotic cell death (type I programmed cell death) and autophagic cell death (type II programmed cell death) as well [13], [14]. Nevertheless, whether autophagy participates antilymphoma antibody-induced cell death has not been identified.

More recently, several signaling pathways like mTOR, PI3K, Akt, Beclin-1 and HIF-1 have been reported to be involved in the regulation of autophagy. Some of those are also linked to cell death or survival [15]. mTOR is one of the most important regulators of autophagy which integrates signals to govern protein biosynthesis, cell cycle progression, and cell growth [16]. mTOR protein is the catalytic subunit of two molecular complexes: mTORC1 and mTORC2. The Rapamycin-sensitive mTOR complex 1 (mTORC1) contains mTOR, the regulatory-associated protein of mTOR (raptor), the proline-rich Akt substrate 40 (PRAS40), mLST8/G-protein b-subunit–like protein (GbL) and deptor, which is regarded as the major part of autophagy regulation [17], [18]. Beclin-1, also known as autophagy-related gene (Atg 6), positively contributes to autophagosome membrane appearance [19], [20]. Beclin-1, together with its binding partner class III phosphoinositide 3-kinase is also required for the initiation of the formation of the autophagosome in autophagy [21]. These signaling pathways are proven to play an important role in Cetuximab-induced cell death [12], [15]. However, signaling pathways of autophagy in chLym-1-induced cell death in lymphoma cells has not been reported yet.

In this paper, we report for the first time that chLym-1 induces autophagy in Raji lymphoma cells. We also investigate the roles of autophagy in chLym-1-induced cytotoxicity, apoptosis, ADCC or CDC. Furthermore, we evaluate the mechanisms of autophagy to mediate apoptosis and the upstream signaling pathways of autophagy as well. Our results highlight a critical indication for enhancing the response of lymphoma cells to chLym-1 through autophagy induction.

## Materials and Methods

### Materials

ChLym-1 was kindly provided by Medipharm Biotech Pharmaceutical (Shanghai, China) and stored at 4°C. Rapamycin, SDS, DMF and NH_4_Cl were purchased by Sangon Biotech Shanghai Co, Ltd. The MEK1/2 inhibitor U0126, and antibodies to LC3, Beta-actin, Phospho-mTOR (Ser2448), Phospho-Akt (Ser473), Phospho-p44/42 MAPK (Erk1/2) (Thr202/Tyr204), and Caspase 9 were obtained from Cell Signaling Technology (Danvers, MA, USA). The antibodies to Phospho-4EBP1 (T45) and Phospho-TSC2 (S939) were obtained from Epitomics (Burlingame, CA, USA). Cyto-ID® Autophagy Detection Kit was obtained from Enzo Life Sciences, Inc (Farmingdale, NY, USA). Annexin V-FITC Apoptosis Detection Kit was purchased from BD Biosciences (Franklin Lakes, NJ, USA). The second-antibodies horseradish peroxidase (HRP)-conjugated goat anti-mouse and anti-rabbit immunoglobulin G (IgG) was obtained from MR Biotech (Shanghai, China). 3-MA was purchased from Sigma (St Louis, MO, USA) and stored at −20°C.

### Cell Culture

Raji Burkitt’s lymphoma cells and Daudi cells were obtained from Cell Bank of Chinese Academy of Sciences, Shanghai Branch (Shanghai, China), and routinely cultured in RMPI1640 supplemented with 10% fetal bovine serum (FBS) and 1% penicillin-streptomycin solution. Cells were maintained at 37°C in environment of 95% air and 5% CO_2_. For experimental use, chLym-1, 3-MA, NH_4_Cl and Rapamycin were prepared and diluted with RMPI1640.

### Immunoblotting Procedures

Raji cells were washed with cold PBS and then lysed in Ripa buffer (Beyotime, Jiangsu, China) for 20 minutes on ice. The lysates were cleared by centrifugation (12000×g, 4°C) for 5 minutes. Protein content was measured by Protein Assay Kit (Beyotime, Jiangsu, China), and 50 µg of protein of each sample was resuspended in 5×loading buffer, resolved by electrophoresis on 10–15% SDS-PAGE, and transferred to PVDF membranes. The PVDF membranes were washed in TBS for 3 times and immediately blocked in TBST containing 5% non-fat milk for 1 h. Then the PVDF membranes were incubated overnight with antibodies (1∶1000 of dilution) in TBST buffer at 4°C with gentle shaking. Then membranes were washed in TBST and hybridized with horseradish peroxidase conjugated anti-rabbit antibody for 1 h, and immunoreactive bands were detected by chemiluminiscence reagent (Pierce Biotechnology, Inc, USA). Experiments involving immunoblotting procedures were repeated at least three times and blots were re-probed with antibodies for β-actin or β-Tublin to control for protein loading and transfer. Densitometric values of proteins bands were quantified using the IQuantTL software (GE Healthcare, England, UK).

### Transmission Electron Microscopy

Raji cells and Daudi were incubated with 10 µg/ml of chLym-1 for 24 h and treated as described [22]. The samples were then stained with uranylacetate and lead citrate in a Leica Ultracut microtome and examined with a JEM-1230 transmission electron microscopy at an accelerating voltage of 60 KV. Digital images were obtained using a specific imaging system.

### Immunofluorescence Confocal Microscopy

Raji cells were seeded at approximately 5,000 cells/well in 96-well clear bottom imaging tissue culture plates (NEST Biotechnology Co., Ltd., Jiangsu, China) and pre-treated as described [23]. Then samples were stained by specific stain with Cyto-ID® Autophagy Detection Kit for immunofluorescence confocal assay [24]. Merged images were obtained according to the Recommended Assay Procedure using AttovisionTM software (Becton, Dickinson and Company, New Jersey, USA).

### siRNA Transfection

siRNA against Atg5 (sense sequence: GUGAGAUAUGGUUUGAAUA; antisense sequence: CACUCUAUACCAAACUUAU), and a negative control siRNA were obtained from GUANGZHOU RIBOBIO CO., LTD (Guangzhou, China). For siRNA transfection, cells (5×10^5^ cells/ml) transfected the siRNA oligonucleotides using X-tremeGENE siRNA Transfection Reagent (Roche Diagnostics, Indianapolis, IN, USA) according to the manufacturer’s instructions, and cultured for 48 h for further treatments or western blot assays.

### MTT-based Cell Viability Assays

Cell viability was determined by using MTT (3–4, 5-dimethylthiazol-2-yl-2, 5-diphenyl-tetrazolium bromide) assay. Raji Cells in exponential growth were seeded into 96-well plates at a concentration of 2×10^5^ cells/ml. ChLym-1 with or without 3-MA, NH_4_Cl, or Rapamycin diluted with RMPI1640 was added to cultures. Autophagy inhibitor 3-MA and NH_4_Cl were added 1 h before chLym-1 treatment. Cells treated with RMPI640 alone were used as a control. Then 10 µl of MTT solution was mixed to each culture after 48 h of co-incubation with antibody and autophagy inhibitor. Cells were maintained at 37°C in environment of 5% CO_2_ for 4 h and 100 µl of lysis solution (20%SDS, 50% DMF) was then added. The optical density (O.D.) values of the Raji cells at wavelength 570 nm were measured to reflect the relative number of surviving cells. The O.D. values of the treated cells were normalized to that of the negative control and expressed as a percentage of the O.D. value of the control.

### Flow Cytometry (FCM) Assay

After incubation with or without 10 µg/ml chLym-1 and autophagy inhibitors for 24 h, Raji cells were stained with Annexin V and PI, and then measured by an apoptosis assay through FCM. Both Annexin V+/PI+ cells and Annexin V+/PI- cells have been considered as apoptotic cells.

### ADCC Assay

The ability of chLym-1 to mediate ADCC after autophagy inhibition was assessed employing human peripheral blood mononuclear cells (PBMC) as effecter cells, and measured by a 5 h LDH release assay [25]. Pretreated with autophagy inhibitor 3-MA and NH_4_Cl for 12 h, Raji cells were incubated with 10 µg/ml of chLym-1 and autophagy suppressor 3-MA or NH_4_Cl in the presence of PBMC at a ratio of 1∶20, 1∶40 or 1∶60 for 5 h. For further confirm the role of autophagy in chLym-1-induced ADCC, siRNA anti-ATG5 was transferred into Raji cells 48 h before ADCC assay. Then cells were incubated with 10 µg/ml of chLym-1 in the presence of PBMC at a ratio of 1∶20, 1∶40 or 1∶60 for 5 h. The release of LDH was measured with cytotoxity 96 kit (Promega BioSciences, LLC., CA, USA). The percent-specific lysis was determined by the following equation: [(E-T)/(H-T)]×100, where E is the mean LDH released in the test samples with effector cells, H is the mean LDH released in the presence of lysis buffer, and T is the mean LDH released by target cells incubated with medium alone [2].

### CDC Assay

After treatment with 3-MA or NH_4_Cl for 24 h, Raji cells were cultured with 10 µg/ml of chLym-1 and 3-MA or NH_4_Cl for 1 h, with 5% human AB serum co-incubation. The ability of chLym-1 to mediate CDC after autophagy inhibition was immediately detected by MTT assay [26].

### Caspase 3/9 Activity Assay

After incubation with 10 µg/ml of chLym-1 and autophagy inhibitors for 24 h, Raji cells were lysed and the activity of apoptosis-related protein Caspase 3 and Caspase 9 were measured by ELISA kits (Beyotime, Jiangsu, China) [27], [28].

### Statistics Analysis

Statistics analysis was carried out with IBM SPSS Statistics 19. The results were expressed as mean ± SD. Comparisons were performed using Student’s *t* test (two-tailed), and One way Anova. *P*-value<0.05 was considered statistically significant.

## Results

### Autophagy is Significantly Induced by chLym-1 in Raji Cells

**Figure 1:**
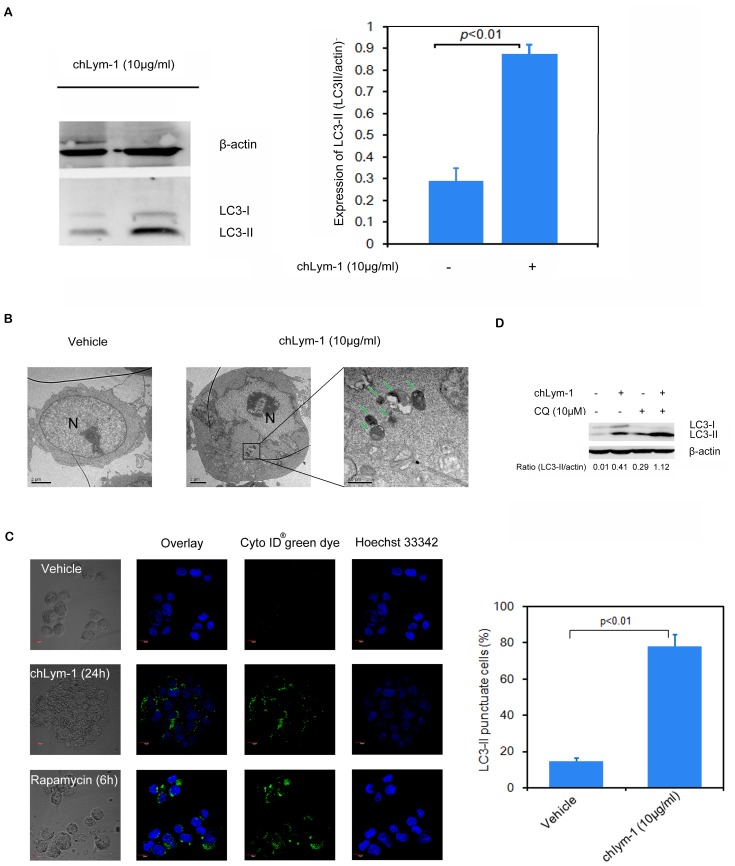
Autophagy could be significantly induced by chLym-1 in Raji cells. A: Autophagy-related protein LC3-II is significantly up-regulated in Raji cells treated with chLym-1. Raji cells were treated with 10 µg/ml of chLym-1 for 24 h as described, while vehicles were treated with complete medium. Statistics was applied to detect relative intensities of LC3-II (LC3-II/actin). B: ChLym-1 induces autophagosomes accumulation (arrows) in Raji cells. Raji cells were treated with/without 10 µg/ml of chLym-1 for 24 h, and then prepared for transmission electron microscope. N = Nuclear. C: Appearance of autophagosome membrane-associated LC3-II in Raji cells treated with chLym-1. Raji cells were treated with/without chLym-1 for 24 h, and vehicles were treated with RMPI1640 supplemented with 10% fetal bovine serum (FBS). Raji cells treated with 50 nM of Rapamycin for 6 h were used as positive control. Spots were quantified by IQuantTL (GE Health Care). D: chLym-1 induces autophagy via autophagosomes accumulation but not via inhibition of autophagosomes degradation. Raji cell were treated with chLym-1 and/or 10 mM of Chloroquine (CQ) for 24 h.

Autophagy can be induced upon chLym-1 treatment in Raji lymphoma cells. The expression of autophagy related protein LC3-II (16 KD) significantly increases in chLym-1-treated Raji cells but not in Daudi cells, which does not combine to chLym-1 (*p*<0.01, Figure 1A, Figure S1,S2). Transmission electron microscopy studies reveal that autophagosomes accumulation in Raji cells after chLym-1 treatment for 24 h, while autophagosomes are scarce in non-treated control cells and chLym-1-treated Daudi cells (Figure 1B, Figure S3). When stained by Cyto-ID® Autophagy Detection Kit, as Rapamycin-treated Raji cells (positive control), cells treated with chLym-1 (10 µg/ml) for 24 h display more punctuate fluorescence (LC3-II) than non-treated cells which show minimal punctuate fluorescence under immunofluorescence confocal microscopy (Figure 1C). Moreover, blocking autophagy by CQ, an autolysosome inhibitor, can additionally enhance the expression of LC3-II in chLym-1-treated Raji cells (Figure 1D), suggesting that chLym-1 induces autophagy via autophagosomes accumulation, but not via inhibition of autophagosomes degradation. Together, our results strongly suggest that chLym-1 can induce autophagy in Raji lymphoma cells.

### Autophagy Inhibitors Suppress chLym-1-induced Cytotoxicity, and Autophagy Inducer Enhances Cell Death of Raji Cells

**Figure 2:**
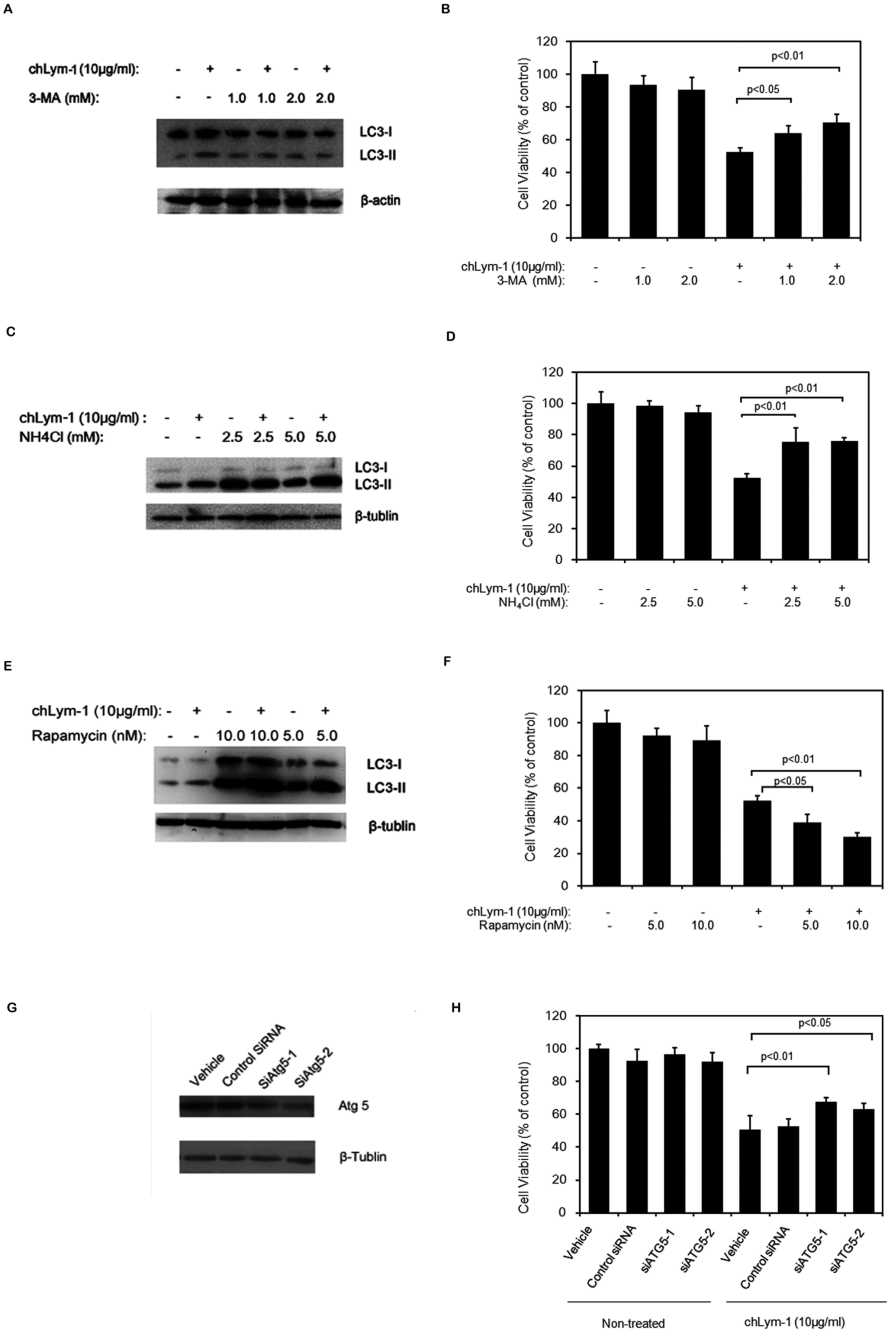
ChLym-1-induced cell death is inhibited through autophgy suppression with PI3K inhibitor 3-MA or lysosome inhibitor NH_4_Cl, and was enhanced with autophagy inducer Rapamycin. A, C: Autophagy inhibitor 3-MA and NH_4_Cl leads to the accumulation of LC3-II and the disappearance of LC3-II induced by chLym-1. Raji cells were treated with 3-MA/NH_4_Cl or/and chLym-1 for 24 h. Then, western blot was used to detect the expression level of LC3-II. B, D: Suppression of autophagy with 3-MA and NH_4_Cl suppresses growth inhibition induced by chLym-1 in Raji Cells. Raji cells were treated with autophagy inhibitors 3-MA/NH_4_Cl or/and chLym-1 for 48 h. The relative number of surviving cells was determined with an MTT assay. E: Autophagy inducer Rapamycin gives rise to the accumulation of LC3-II. Raji cells were treated with autophagy inducer Rapamycin or/and chLym-1 for 24 h. Then, western blot was used to detect the expression level of LC3-II. F: Enhancement of autophagy with Rapamycin promoted growth inhibition induced by chLym-1 in Raji Cells. Raji cells were treated with autophagy inducer Rapamycin or/and chLym-1 for 48 h. The relative number of surviving cells was determined with an MTT assay. G: Anti-ATG5 siRNA suppresses ATG5 expression. Raji cells were transferred with/without siRNA targeting control, ATG5-1 and ATG5-2 for 48 h, then western blot was used to detect the expression level of ATG5. H: Inference of ATG5 significantly rescued viability of Raji cells treated with chLym-1. Raji cells were transferred with/without siRNA targeting control, ATG5-1 and ATG5-2 for 48 h, then co-incubated with chLym-1 for 48 h. The relative number of surviving cells was determined with an MTT assay.

Raji cells treated with chLym-1 (10 µg/ml) for 48 h show a 50% inhibition of cell viability when compare with non-treated Raji cells (Figure 2B, D, and F). 3-MA inhibits autophagy through type III PI3K suppression and has no effect on p-AKT-S473 at 2 mM (Figure S4), which mediates the decrease of autophagosomic form of LC3 (LC3-II) (Figure 2A), while NH_4_Cl suppresses degradation of lysosomes and elevates autophagosomic form of LC3 (LC3-II) (Figure 2C). Rapamycin-induced LC3-II is caused by the suppression of mTOR pathway, which further promotes additional induction of autophagy in Raji cells (Figure 2E). Figure 2B and 2D also reveal that compared with Raji cells treated with chLym-1 alone, Raji cells treated with chLym-1 in combination with autophagy inhibitor 3-MA and NH_4_Cl show a significant rescue of cell viability after 48 h of co-incubation, while 3-MA and NH_4_Cl have no significant effect on viability of Raji cells. (*p*<0.05, *p*<0.01) (Figure 2B, D); However, the viability of Raji cells treated with chLym-1 and Rapamycin is significantly inhibited compared with that of Raji cells treated with chLym-1 alone (Figure 2F). Moreover, compared to non-inference Raji cells, cells transferred with control siRNA did not rescue the viability of Raji cells, while the tumor-killing effect of chLym-1 on ATG-5-knockdown Raji cells has been decreased (*p*<0.05, *p*<0.01) (Figure 2 G, H).

Taken together, these data strongly indicate that suppression of autophagy induced by chLym-1 blocks chLym-1-induced cytotoxicity of Raji cells and additional induction of autophagy by Rapamycin enhances cytotoxicity, which indicates that autophagy played a critical role in chLym-1-induced cytotoxicity of Raji cells.

### Suppression of Autophagy Inhibits chLym-1-induced Apoptosis, ADCC and CDC of Raji Cells

**Figure 3:**
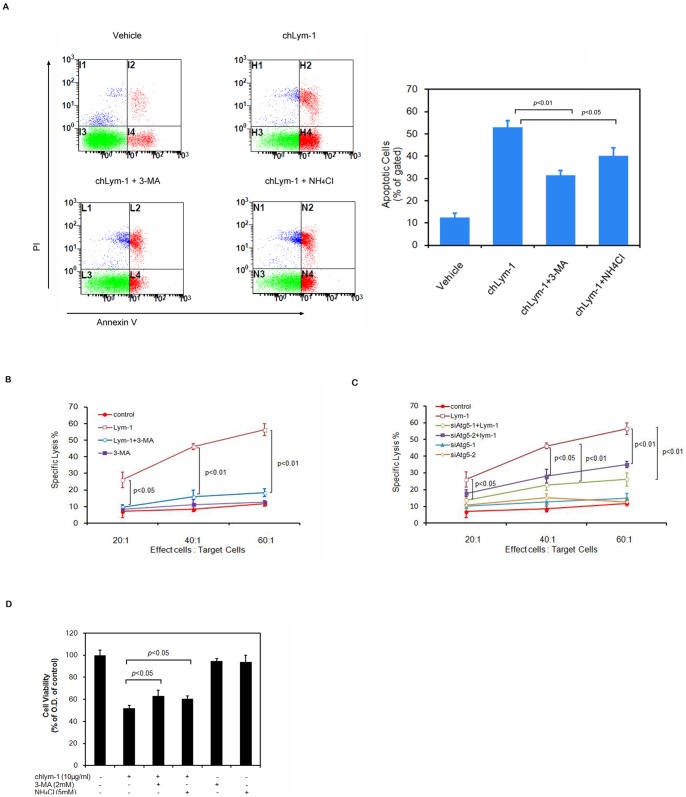
Inhibition of autophagy with PI3K inhibitor 3-MA and lysosome inhibitor NH_4_Cl suppress chLym-1induced apoptosis, antibody-dependent cellular cytotoxicity(ADCC) and complement-dependent cytotoxicity(CDC) in Raji cells. A: 3-MA and NH_4_Cl significantly inhibit apoptosis induced by chLym-1 in Raji cells. Raji cells were treated with 10 µg/ml of chLym-1 or/and autophagy inhibitors for 24 h, while Vehicles were treated with full medium. Thus, FCM was used to detect apoptotic rates of Raji cells. B: ChLym-1-induced ADCC is inhibited by PI3K inhibitor 3-MA. Raji cells, together with PBMCs (effect cells: target cells = 60∶1, 40∶1 and 20∶1), were treated with autophagy inhibitor 3-MA or/and chLym-1 for 5 h. Autophagy inhibitor 3-MA was added 12 h before chLym-1 treatment. Then, ADCC mediated by chLym-1 on Raji cells was measured in a 5 h LDH release assay. C: ChLym-1-induced ADCC is inhibited by Anti-ATG5 RNA inference. Raji cells (non-treated, transferred with control siRNA or transferred with Anti-ATG5 siRNA), together with PBMCs (effect cells: target cells = 60∶1, 40∶1 and 20∶1), were treated with chLym-1 for 5 h. ATG5 RNA-inference is performed 48 h before chLym-1 treatment. Then, ADCC mediated by chLym-1 on Raji cells was measured in a 5 h LDH release assay. D: ChLym-1 mediated CDC is blocked by PI3K inhibitor 3-MA or lysosome inhibitor NH_4_Cl. Raji cells were treated with autophagy inhibitor 3-MA/NH_4_Cl or/and chLym-1 for 1 h. Autophagy inhibitor 3-MA and NH_4_Cl was added 24 h before chLym-1 treatment. Then, CDC mediated by chLym-1 on Raji cells was measured immediately by MTT assay.

Monoclonal antibody could induce apoptosis, ADCC and CDC to kill tumor cells, which is known to be associated with autophagy. The results of FCM analysis based on Annexin V and PI stain assays of Raji cells show that 24 h-treatment with chLym-1 induces notable apoptosis of Raji cells, which is suppressed by 3-MA and NH_4_Cl (Figure 3A). Moreover, the ability of chLym-1 to mediate ADCC and CDC was analyzed after autophagy inhibition with 3-MA and NH_4_Cl. Our findings show that 10 µg/ml of chLym-1 has no significant effect on viability of PBMCs (Figure S5) but induces remarkable ADCC, causing obvious cell lysis of Raji cells, which was significantly rescued by PI3K inhibitor 3-MA and ATG5-knockdown (Figure 3B, 3C), whereas 5 mM of lysosome inhibitor NH4Cl does not contribute to cell survival in Raji cells (data not shown). In CDC assays, our data reveal that 10 µg/ml of chLym-1 mediates notable CDC to kill Raji cells by co-incubating with human AB serum, which can be partly blocked by autophagy inhibitors 3-MA and NH_4_Cl (Figure 3D).

Together, these data strongly suggest that inhibition of autophagy could block chLym-1-induced apoptosis, ADCC and CDC, further demonstrating that autophagy played an important role in chLym-1-induced apoptosis, ADCC and CDC.

### ChLym-1 Mediates Caspase-dependent Apoptosis via Autophagy Induction

**Figure 4:**
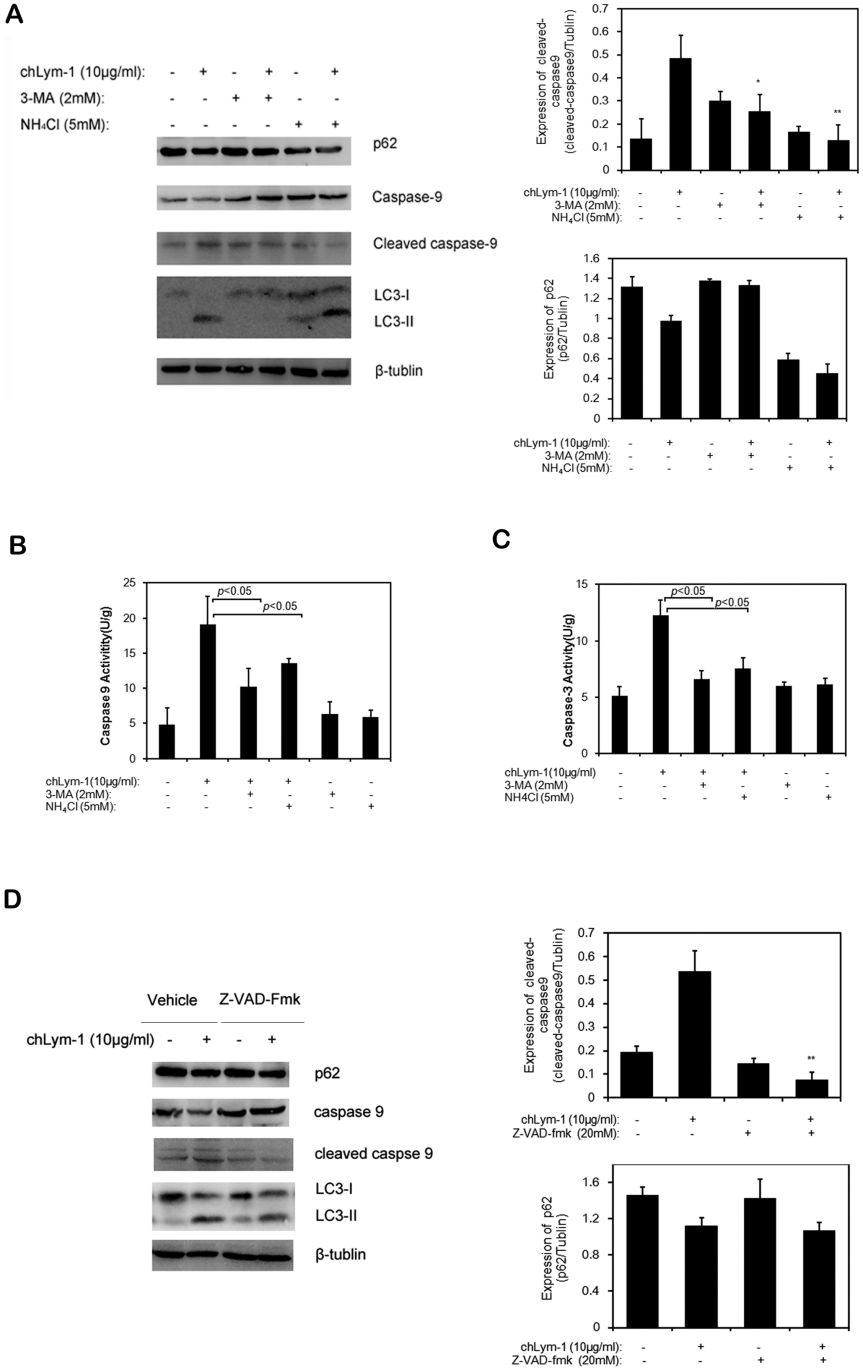
chLym-1 mediates caspase-dependent apoptosis via autophagy induction. A: Autophagy inhibitors significantly inhibit caspase 9 activation. Raji cells Raji cells were treated with autophagy inhibitor 3-MA/NH_4_Cl or/and chLym-1 for 24 h. Autophagy inhibitor 3-MA and NH_4_Cl was added 1 h before chLym-1 treatment. Then, western blot was used to detect expression of LC3-II and caspase 9 in Raji cells. Statistics was applied to detect relative intensities of cleaved-caspase 9 and p62. *p<0.05, **p<0.01 v.s. chLym-1. B, C: Inhibition of autophagy with 3-MA and NH_4_Cl significantly reduce the activity of caspase 3 and caspase 9. Caspase 3 and Caspase 9 assay were measured after 24 h of incubation of Raji cells treated with autophagy inhibitors or/and chLym-1. D: Inhibition of caspase 9 activation has no effects on expression of autophagy related protein LC3-II and p62. Raji cells were treated with either or both apoptosis inhibitor z-VAD-fmk and chLym-1 for 24 h. Then, western blot was used to detect LC3-II, p62 and caspase 9 expression of Raji cells. Statistics was applied to detect relative intensities of cleaved-caspase 9 and p62. **p<0.01 v.s. chLym-1.

In the previous study, chLym-1 could induce caspase-dependent apoptosis in lymphoma cells. Caspase 3 and Caspase 9 were measured after autophagy inhibitor treatment. Caspase 9 is cleaved in chLym-1 treated Raji cells but remains as uncleaved type in autophagy inhibitor treated Raji cells (Figure 4A). Furthermore, we find that Caspase 9 and Caspase 3 are activated after chLym-1 treatment for 24 h, but are inactivated under the effect of 3-MA and NH_4_Cl (Figure 4B, C). At last, the expression of LC3-II and p62 were detected under the effect of z-VAD-fmk, a caspase inhibitor, revealing that z-VAD-fmk suppressed the activation of Caspase 9 but had no effect on LC3-II and p62 (Figure 4D).

Together, our data indicate that chLym-1 mediates caspase-dependent apoptosis via autophagy induction, which further demonstrate the role of autophagy in chLym-1-induced apoptosis.

### ChLym-1 Activated Akt/mTOR Pathway and MEK/Erk Pathway in Autophagy Induction of chLym-1 in Raji Cells

**Figure 5:**
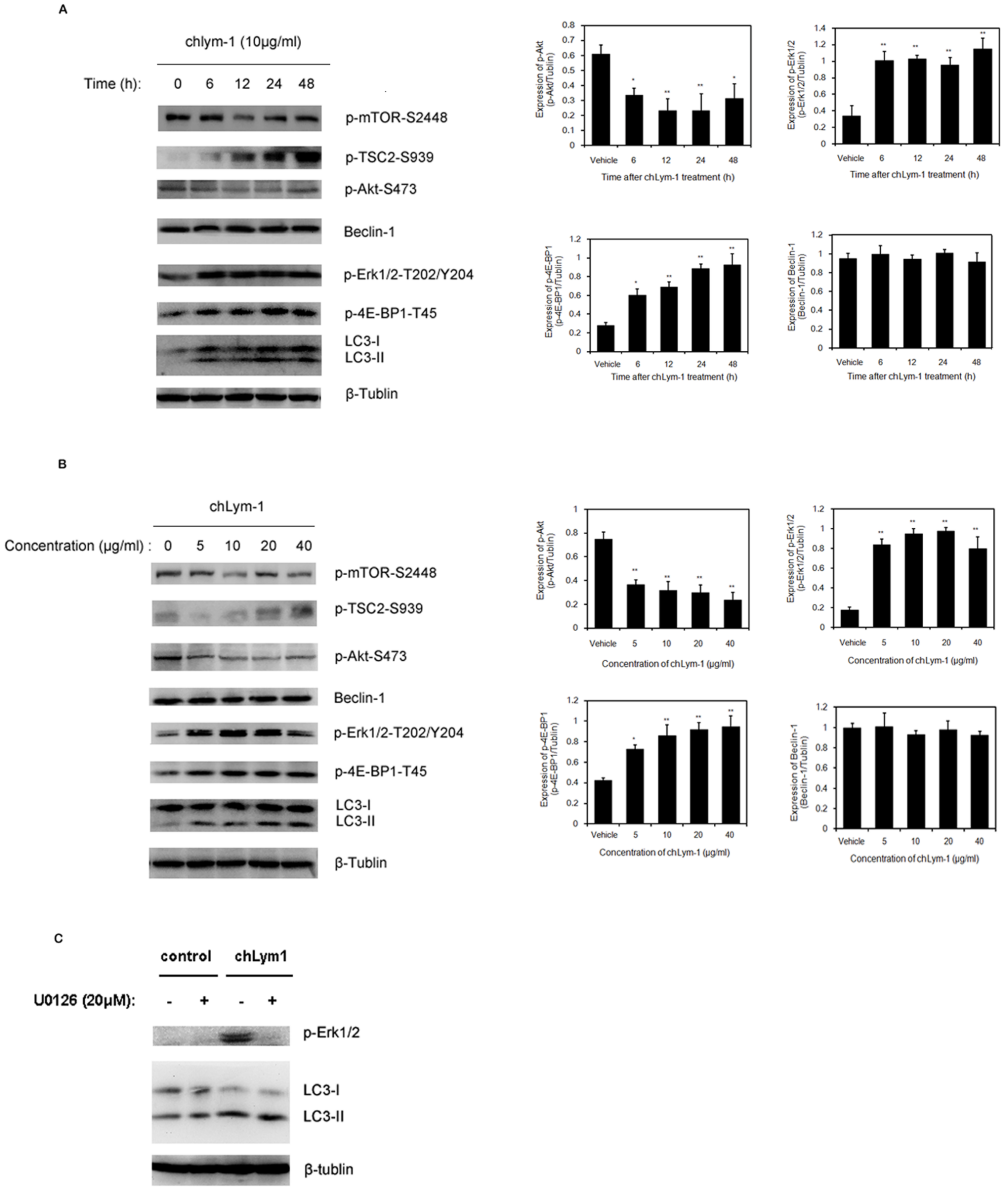
chLym-1 activates Akt-mTOR pathway, MEK-Erk1/2 pathway and induces autophagy in Raji cells. A, B: chLym-1 treatment leads to the p-Akt and p-mTOR inhibition, and phosphorylation of 4E-BP1, Erk1/2 and TSC2 are up-regulated. Raji cells were treated with chLym-1 in different concentrations and for different times. Then, western blot were used to observe the expression of autophagy related proteins. Statistics was applied to detect relative intensities of p-Akt-S473, p-Erk1/2-T202/Y204 and Beclin-1. *p<0.05, **p<0.01 v.s. vehicle. C: U0126 does not block the expression of LC3-II in Raji cells. Raji cells were treated with either or both MEK-Erk1/2 inhibitor U0126 and chLym-1 (10 µg/ml) for 24 h. Then, western blot were used to observe the expression of LC3-II.

For further research of the mechanism of chLym-1-induced autophagy, several upstream signaling pathways were measured after chLym-1 treatment. Akt/mTOR pathway is one of the most important regulatory pathways of autophagy, which is down-regulated in chLym-1-treated Raji cells. As autophagosomic form of LC3 (LC3-II) increased in Raji cells treated with chLym-1 in different concentrations or for different time, p-Akt and p-mTOR are inhibited whereas phosphorylation of 4E-BP1 and TSC2 are up-regulated, indicating the inactivation of Akt/mTOR pathway (Figure 5A, B; Figure S6,S7). In addition, phosphorylation of Erk1/2 is observed to be up-regulated in chLym-1 treated Raji cells whereas the expression of beclin-1 remains no change (Figure 5A, B), suggesting that MEK-Erk1/2 pathway but not beclin-1 is activated through autophagy induction of chLym-1. Moreover, blocking Erk1/2 with U0126 does not affect the expression of autophagosomic form of LC3 (LC3-II) of chLym-1-treated Raji cells (Figure 5C).

All these data suggest that chLym-1 activate Akt/mTOR pathway and MEK-Erk1/2 pathway. Akt/mTOR pathway is most likely to participate in autophagy induction of chLym-1.

## Discussion

HLA-DR is high expressed in lymphocytes and lymphoma cells such as SU-6-DHL, B-35M and Raji cell. As an anti-HLA-DR monoclonal antibody, chLym-1 shows its high efficiency in lymphoma trails whereas the exact mechanism still remained unclear [29]. As a critical way for programmed cell death, autophagy leads to cell death in some circumstances. Many studies revealed that inhibition of mTOR complex and enhancement of autophagy involved in growth inhibition, apoptotic cell death in lymphoma cells and may correlates with great clinical outcome, suggesting a tumor-suppression role of autophagy in Non-Hodgkin’s lymphoma treatment [30]–[33]. In this paper, for the first time, we report that chLym-1 can induce autophagy in lymphoma cells and investigate the role and the mechanism of autophagy in chLym-1-induced apoptosis, ADCC and CDC.

We used several standard experimental techniques to evaluate the chLym-1 inducing autophagy in Raji cells, including fluorescent microscopy, western blot and transmission electron microscopy, showing more punctuate fluorescence (LC3-II), appearance of membrane-associated protein-LC3-II and the formation of characteristic autophagosomes after 24 h-treatment with chLym-1. All these evidences strongly suggest that autophagy is induced by chLym-1 in Raji cells [34].

In order to investigate the role of autophagy in Raji cells treated with chLym-1, we used MTT assay for the cell viability test and western blot to determine the expression of LC3-II. First of all, the results of western blot show that autophagy inhibitor NH_4_Cl could suppress the chLym-1-induced autophagic flux, leading to the accumulation of LC3-II; and autophagy inhibitor 3-MA could block PI3KC3, giving rise to the disappearance of LC3-II induced by chLym-1 [12], [35]. However, Rapamycin induces autophagic flux through mTOR inactivation, also resulting in the accumulation of LC3-II [12], which indicates the autophagy activation of Raji cells. In addition, MTT assays show that inhibition of autophagy with 3-MA and NH_4_Cl could partly block cytotocixity caused by chLym-1, while enhancement of autophagy by Rapamycin facilitates cell death of Raji cells. Together, our results indicate that autophagy plays a tumor-suppressing role in chLym-1-induced cytotoxicity.

One of the most important points in this work is that autophagy is involved in chLym-1-induced apoptosis, ADCC and CDC. Firstly, the results of apoptosis assays based on FCM show that 3-MA and NH_4_Cl could decrease the numbers of Annexin V positive cells, recuing chLym-1-treated Raji cells from apoptosis. Secondly, autophagy suppression in Raji cells also prevents cells from lysis, inhibiting the activity of chLym-1-induced ADCC and CDC. Our data strongly suggest that autophagy participates chLym-1-induced apoptosis, ADCC and CDC.

We further investigated the mechanism of autophagy in apoptosis induction in Raji cells. Western blot studies reveal that Caspase 9 is inactivated through autophagy inhibition with 3-MA and NH_4_Cl. Furthermore, data of activity of Caspase 3 and Caspase 9 exhibit that 3-MA and NH_4_Cl could suppress Caspase 3 and Caspase 9 activation, indicating that autophagy induced by chLym-1 mediate apoptosis through caspase-dependent pathway. At last, we found that inhibition of apoptosis by z-VAD-fmk had no effect on autophagy, indicating that autophagy is not a downstream response to apoptosis in chLym-1-treated Raji cells. Together, our data suggest that chLym-1 can induce caspase-dependent apoptosis via autophagy induction.

Many upstream signaling pathways of autophagy play key roles in autophagy induction and regulation. Western blot studies show the down-regulation of p-Akt and p-mTOR expression and up-regulation of p-TSC2 and p-4EBP1, strongly indicating the activation of Akt-mTOR pathway [36], [37]. In addition, Erk1/2 is activated by phosphorylation in response to chLym-1 induction. However, the expression of LC3-II is reduced when Erk1/2 phosphorylation is not inhibited by U0126, suggesting MEK-Erk1/2 pathway is not involved in autophagy induction of chLym-1 [38], [39]. Moreover, the expression of beclin-1 does not change in Raji cells [40]. Together, our data indicate that several autophagy-related signaling pathways are activated through chLym-1 treatment, and Akt-mTOR pathway is most likely to play an important role in autophagy induction.

Recently, several studies demonstrated that autophagy was significantly associated with cell survival and therapy resistant [8], [12], [41]. However, it is also reported that autophagy could be the mechanism that led to cell death in tumor therapy [13]. AS_2_O_3_ is a typical example supporting this conclusion. As was reported, the autophagy induced by AS_2_O_3_ could mediate programmed cell death II [42], [43]. Also, autophagy could lead to apoptosis as well [44].

In conclusion, we demonstrate that chLym-1 could significantly induce apoptosis-independent autophagy in lymphoma Raji cells. Importantly, chLym-1 has been proved to induce apoptosis, ADCC and CDC via autophagy induction, causing cell cytotoxicity. This indicates that autophagy induced by chLym-1 elicits a tumor suppression mechanism in Raji cells. Moreover, several upstream signaling pathways of autophagy like Akt/mTOR, and MEK/Erk are activated after chLym-1 treatment. Our data also highlights that autophagy induction probably gives rise to the sensitiveness enhancement of lymphoma cells to chLym-1, indicating the potential efficacy enhancement of chLym-1 by autophagy induction in lymphoma therapy.

## Supporting Information

Figure S1 **Daudi cell is insensitive to chLym-1 treatment.** Daudi cells were treated with 10 µg/ml of chLym-1 for 12, 24, 48 and 72 h. The relative number of surviving cells was determined with an MTT assay.
(TIF)

Figure S2 **ChLym-1 can not induce accumulation of the membrane form of LC3 (LC3-II) in Daudi cells.** Daudi cells were treated with 10 µg/ml of chLym-1 for 48 h. Statistics was applied to detect relative intensities of LC3-II
(TIF)

Figure S3 **ChLym-1 does not induce autophagosomes accumulation in Daudi cells.** Daudi cells were treated with 10 µg/ml of chLym-1 for 48 h and were immediately prepared for transmission electron microscope. N = Nuclear.
(TIF)

Figure S4 **2** **mM of 3-MA does not affect p-Akt-S473, but significantly reduced the expression of Vps34.** Raji cells were treated or untreated with chLym-1 and/or 3-MA for 48 h. Then, expression of p-Akt-S473 and Vps34 was determined by western blot.
(TIF)

Figure S5 **chLym-1 has no effect on PBMCs.** PBMCs were incubated with chLym-1(10 µg/ml) for 5 h. The relative number of surviving cells was determined with an MTT assay.
(TIF)

Figure S6 **Statistics of relative intensities of p-mTOR and p-TSC2 of Raji cells treated with chLym-1 in time-manner.** *p<0.05, **p<0.01 v.s. vehicle.
(TIF)

Figure S7 **Statistics of relative intensities of p-mTOR and p-TSC2 of Raji cells treated with chLym-1 in dose-manner.** *p<0.05, **p<0.01 v.s. vehicle.
(TIF)
