# Supplementary figures and images for: Autophagy Plays a Critical Role in ChLym-1-Induced Cytotoxicity of Non-Hodgkin’s Lymphoma Cells
Source: PLoS One. 2013 Aug 28;8(8):e72478. doi: 10.1371/journal.pone.0072478 (PMC3756084; doi:10.1371/journal.pone.0072478)

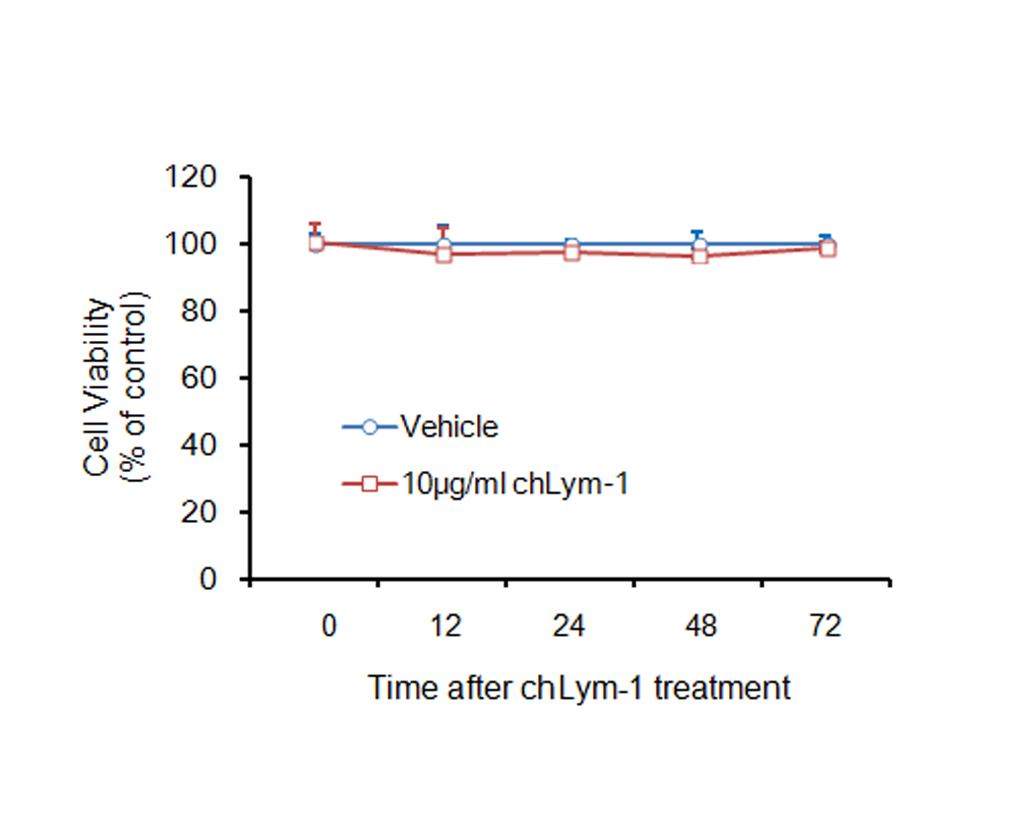

Supplement: Figure S1 — Daudi cell is insensitive to chLym-1 treatment. Daudi cells were treated with 10 µg/ml of chLym-1 for 12, 24, 48 and 72 h. The relative number of surviving cells was determined with an MTT assay. (TIF) [file pone.0072478.s001.tif]

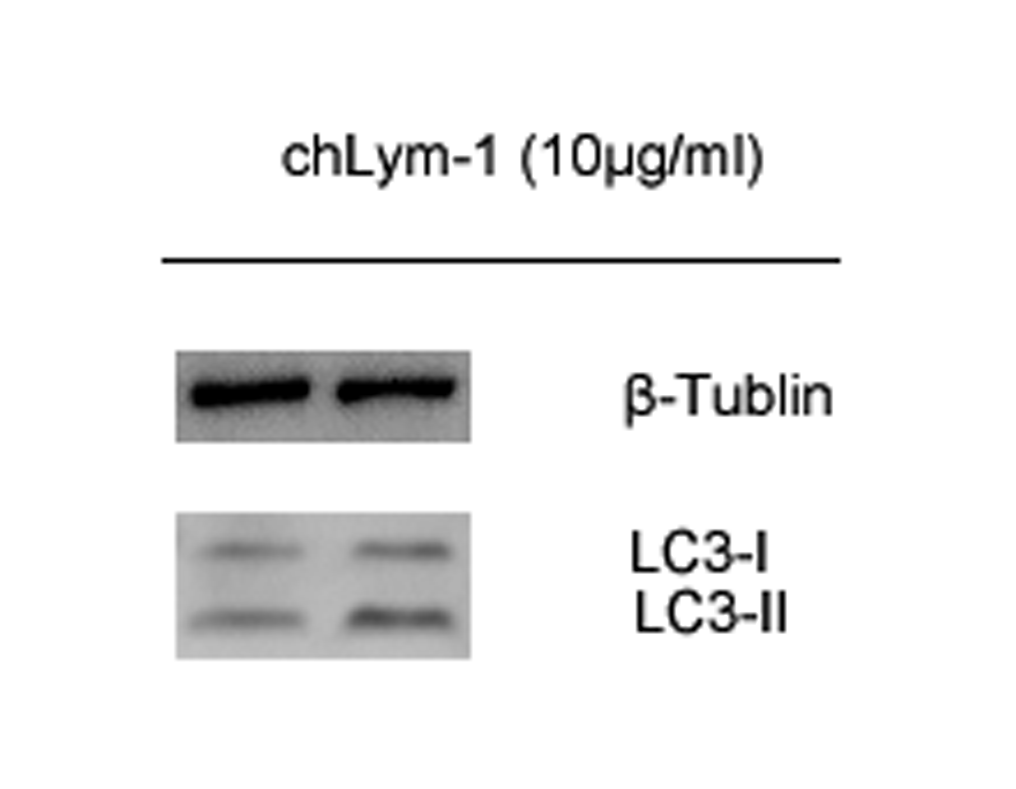

Supplement: Figure S2 — ChLym-1 can not induce accumulation of the membrane form of LC3 (LC3-II) in Daudi cells. Daudi cells were treated with 10 µg/ml of chLym-1 for 48 h. Statistics was applied to detect relative intensities of LC3-II (TIF) [file pone.0072478.s002.tif]

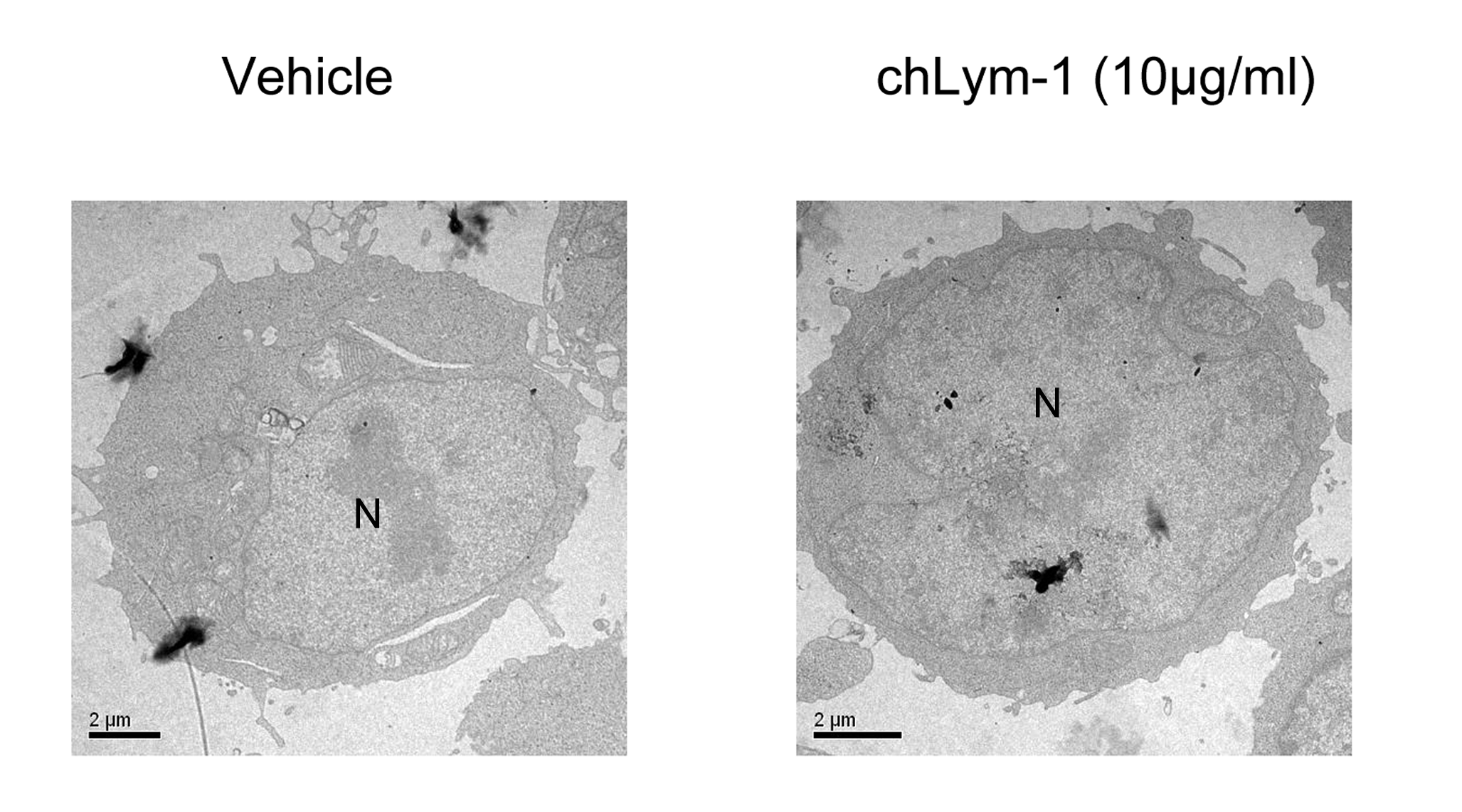

Supplement: Figure S3 — ChLym-1 does not induce autophagosomes accumulation in Daudi cells. Daudi cells were treated with 10 µg/ml of chLym-1 for 48 h and were immediately prepared for transmission electron microscope. N = Nuclear. (TIF) [file pone.0072478.s003.tif]

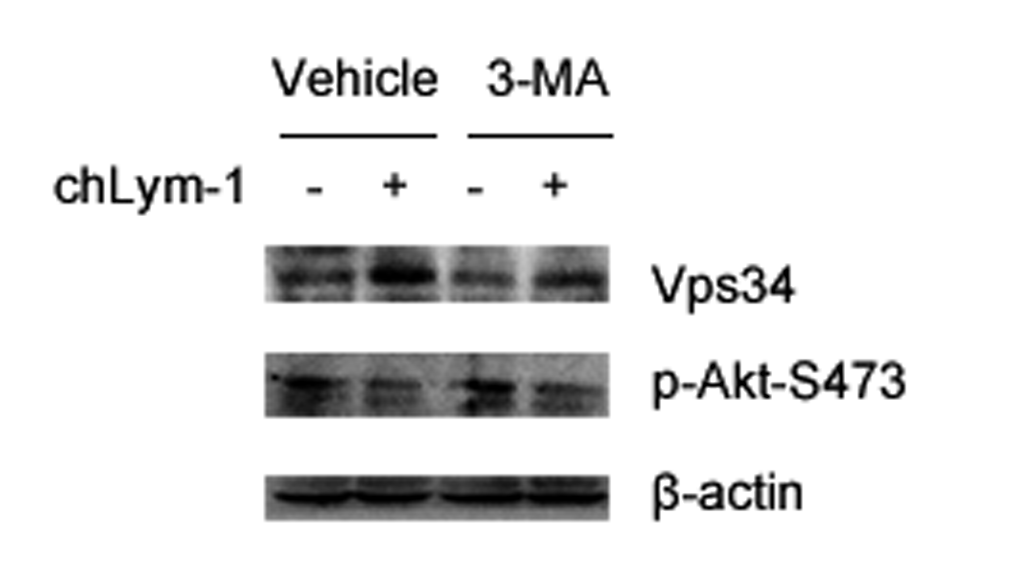

Supplement: Figure S4 — 2 mM of 3-MA does not affect p-Akt-S473, but significantly reduced the expression of Vps34. Raji cells were treated or untreated with chLym-1 and/or 3-MA for 48 h. Then, expression of p-Akt-S473 and Vps34 was determined by western blot. (TIF) [file pone.0072478.s004.tif]

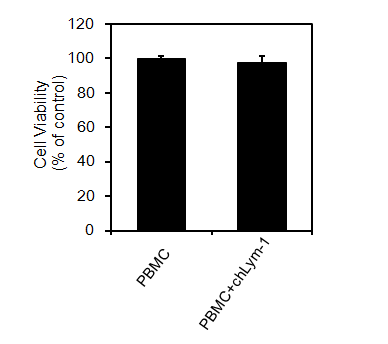

Supplement: Figure S5 — chLym-1 has no effect on PBMCs. PBMCs were incubated with chLym-1(10 µg/ml) for 5 h. The relative number of surviving cells was determined with an MTT assay. (TIF) [file pone.0072478.s005.tif]

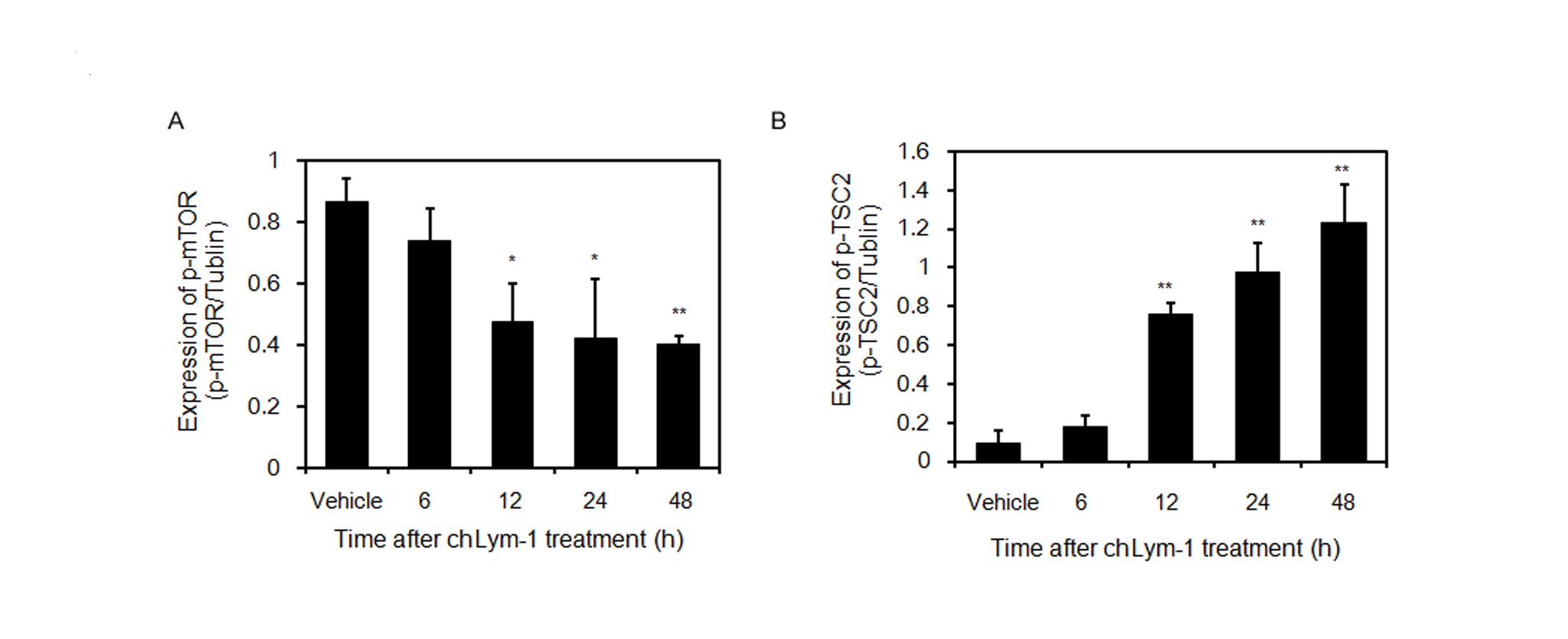

Supplement: Figure S6 — Statistics of relative intensities of p-mTOR and p-TSC2 of Raji cells treated with chLym-1 in time-manner. *p<0.05, **p<0.01 v.s. vehicle. (TIF) [file pone.0072478.s006.tif]

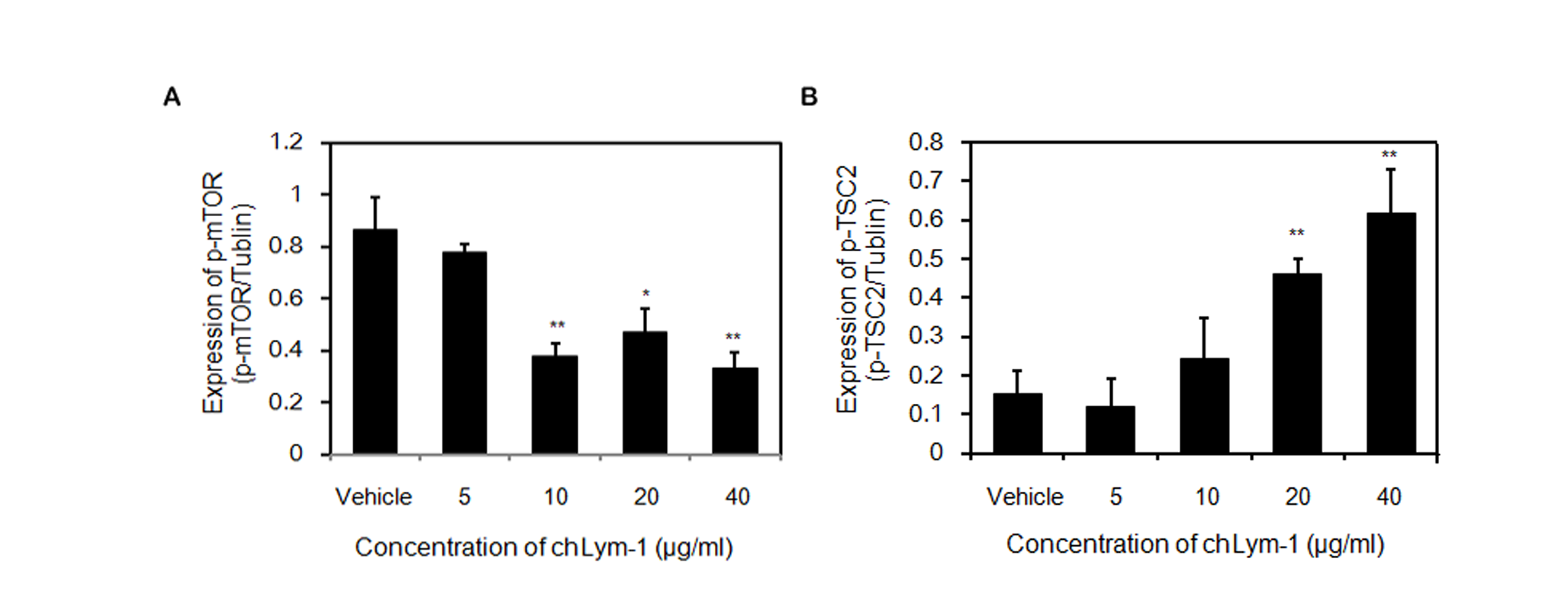

Supplement: Figure S7 — Statistics of relative intensities of p-mTOR and p-TSC2 of Raji cells treated with chLym-1 in dose-manner. *p<0.05, **p<0.01 v.s. vehicle. (TIF) [file pone.0072478.s007.tif]
